# Developing recommendations for promoting wellbeing in individuals with alopecia areata: a modified Delphi study

**DOI:** 10.1136/bmjopen-2024-094491

**Published:** 2026-02-10

**Authors:** Fabio Zucchelli, Kerry Montgomery

**Affiliations:** 1 University of the West of England, Bristol, UK; 2 Centre for Appearance Research, UWE Bristol, Bristol, UK

**Keywords:** Delphi Technique, DERMATOLOGY, Psychological Stress, Protocols & guidelines

## Abstract

**Abstract:**

**Objectives:**

Develop recommendations for multidisciplinary, multisector care providers involved in supporting individuals with alopecia areata (AA) to promote their well-being of these individuals. AA is a condition that causes hair loss on the scalp and, for some, the head or whole body and is associated with difficulties in psychosocial adjustment.

**Design:**

A modified Delphi consensus study with three rounds: round 1 was a qualitative survey to generate recommendations; round 2 involved a rating survey to work towards consensus on important items to retain and round 3 asked panellists from four different support roles to establish the most relevant items for their respective roles.

**Setting:**

The UK, across healthcare, charitable and private health and mental health sectors.

**Participants:**

Panellists held two forms of expertise. One group consisted of experts in support roles, comprising medical professionals (general practitioners and dermatologists), mental health professionals, peer facilitators and trichologists. All were selected due to their experience of working with individuals who have AA. The other group consisted of experts by experience, namely adults living with AA who had some experience of receiving support from the above care providers. 48 panellists contributed to round 1 (22 experts by support role, 29 experts by experience and 3 with dual roles), 46 to round 2 (21 experts by support role, 27 experts by experience and 3 with dual roles) and 23 experts by support role completed round 3.

**Data analysis:**

In round 1, data were analysed using qualitative content analysis. In round 2, panellists rated the importance of all recommendation items on a single 1–5 scale. Consensus was determined by ≥80% agreement between panellists that items were moderately or very important.

**Results:**

Multiple candidate recommendations were generated from round 1, and following round 2, all but one were retained in the list presented in round 3. Items rated as relevant to all care providers in round 3 included ‘Validate (and explore) the emotional impact of AA’, ‘Respect and work with individuals’ chosen coping strategies (where no clear harm is caused)’ and ‘There should be a holistic, multi-support-role and multisector approach to psychological support’. There was notable overlap in the recommendation principles generated across each support role, but the details of how these can be delivered by each group differed. There were also a number of unique recommendations for each support role.

**Conclusions:**

Medical professionals, mental health professionals, trichologists and peer support facilitators can each play a role in promoting the psychological well-being of individuals with AA. The distinct roles and skill sets of each group point to the need for a multidisciplinary approach to supporting the well-being of affected individuals.

**STRENGTHS AND LIMITATIONS OF THIS STUDY**

- Included individuals with alopecia areata (AA) as valuable experts by experience in the development of recommendations and used patient and public involvement throughout.
- Addressed the need for all care providers involved in the care of individuals with AA to promote their well-being.
- Included an extensive qualitative survey in the first round to generate meaningful items for the panellist group, while also drawing on extant literature to inform the analysis.
- Underrepresented Black and Asian individuals, as well as men, in the panellist group.
- Addressed the well-being needs and preferences of children and young people with AA to a lesser extent than adults.

## Introduction

 Alopecia areata (AA) is an immune-mediated follicular condition that causes hair loss, with an estimated cumulative lifetime incidence of approximately 2%.^1^ The peak onset age is 25–29 years,^2^ but it can occur at any age, including during childhood. Hair loss from AA typically begins with small patches on the scalp but can progress to full hair loss on the scalp (alopecia totalis) and body (alopecia universalis).

Although around two-thirds of individuals with AA will experience spontaneous full hair regrowth within 5 years, the clinical course for many with AA involves cycles of unpredictable hair loss, regrowth and/or recurrence.^3^ Effective medical treatment has historically been lacking,^4^ though recent advances offer promise of greater effectiveness.^5^

For many, living with AA presents a challenging psychological adjustment. Compared with the general population, adults with AA are around three times more likely to report depression and anxiety symptoms,^6 7^ and limited research indicates children with AA may also be more likely to experience psychological difficulties.^7^ Lower health-related quality of life has also been reported in individuals with AA compared with matched controls.^8^,^10^

The most commonly cited mediator of these difficulties is a change in physical appearance, which can attract unsolicited social attention and cause a shock to self-identity.^11^ Indeed, fear of negative social evaluation and fears concerning social interaction have been reported as the most affected areas of quality of life, indicative of pronounced appearance-focused concerns.^10^ The often unpredictable prognosis of AA can also cause difficulties in adjusting to the condition.^12^

While the psychological impact of AA is well established, there is less empirical direction for promoting psychological well-being in this group. This is a particularly important endeavour given that in the UK, many individuals with AA report wanting more psychological support and are dissatisfied with existing provision in the National Health Service.^13^ In terms of intervention approaches, two small-sample studies show promise for mindfulness-based interventions^14 15^ and two for clinical hypnosis^16 17^ in adults with AA.

However, it is important to view wellbeing promotion as a holistic responsibility across the broad range of care providers with whom individuals with AA engage, beyond mental health professionals whose availability is often limited in health services.^18^ In the healthcare setting, this typically involves general practitioners (GPs) and dermatologists, as well as auxiliary private support from trichologists. Individuals with AA report that consultations with GPs and dermatologists can impact their wellbeing in either direction, attributed in part to either validating or dismissive interactions (eg, conveying ‘it’s only hair’).^13^ In community settings, many people also access private mental health professionals, as well as peer support from organisations and charities such as Alopecia UK, who provide organised group peer support led by peer facilitators. Qualitative research suggests that women who used an online peer support forum found the process validating and normalising, though they also reported unhelpful sharing of inaccurate health information.^19^ To our knowledge, however, there is no published research on in-person peer support for AA. Currently, no systematically developed recommendations exist for promoting wellbeing in individuals with AA for any of these roles.

To better understand how to promote wellbeing in individuals with AA, we can build on existing, predominantly qualitative literature indicating unique considerations for the condition. For example, we know that the sociocultural meanings ascribed to hair are profound and vary across cultures and ethnicities, and its loss can impact the social roles and capital of the affected individuals.^11^ People with AA also navigate decisions concerning concealing or camouflaging hair (eg, through wigs and headwear) as well as associated products and services.^20^ Equally, choosing whether and how to disclose their AA can present individuals with challenges.^21^ Disruptive changes to self-identity and perceived attractiveness are also common.^11 22^

Building on existing literature, this study aims to develop actionable recommendations for all care providers to promote psychological wellbeing in individuals with AA. Rather than focusing on specific psychological approaches or techniques, we sought to develop recommendations spanning guiding principles, such as awareness and understanding of the key psychological considerations for people with AA, approaches to communication and the structure of support. To achieve this aim, we used a modified Delphi consensus design. Through its systematic yet flexible methodology, the Delphi method can help gather information from individuals who hold relevant knowledge and experiences to shape practical guidelines.^23^ The importance of lived experience as relevant knowledge and experience is also being increasingly acknowledged^24 25^; therefore, in this study, we included individuals with AA as experts by experience, alongside care providers.

## Methods

### Patient and public involvement (PPI)

Three individuals with AA helped shape the research question before commencing the presented work. All were female; one was of Black British ethnicity and two were of white British ethnicity. A key way in which this group influenced the research question was by adding GPs and dermatologists (ie, medical professionals) to the scope of care providers who can promote psychological well-being and, hence, use the recommendations.

Upon study commencement, two of these individuals acted as PPI advisors. At every stage of the study, they advised on the methods to be used and reviewed all study materials. They also piloted all relevant surveys. After study completion, the PPI advisors helped shape the dissemination of the recommendations.

In addition to the PPI advisors, the second author contributed as a stakeholder advisor representing the care providers (experts by support role). The second author previously worked as a psychological well-being lead for Alopecia UK and is an academic researcher. Collectively, the above three individuals are henceforth described as *study advisors*. All were female and white British.

### Study protocol

The study commenced with a systematic literature review of the relevant literature to inform the analysis of the first Delphi round. Panellists for the Delphi study were selected through two sources of expertise: expertise by support role (medical professionals, mental health professionals, peer supporters and trichologists) and expertise by experience (individuals with AA).

Round 1 comprised a qualitative survey completed by panellists to generate candidate recommendation items. In round 2, panellists were sent all the items and asked to rate their importance, rank them within their categories and provide qualitative feedback on the items. Items meeting a threshold level of agreement were to be retained in a shortlist. The remaining items were to be sent to the panellists in round 3, with anonymised consensus statistics, inviting panellists to re-rate the items to establish a final consensus. The revised ratings would then be used to produce a final version of recommendations for dissemination to care providers.

Full details of the design, including modifications to the protocol for round 3, are provided in the following sections. The protocol was not registered.

### Literature review

In February 2023, the first author conducted a systematic search of relevant literature. The databases CINAHL Plus, Embase, MEDLINE, PsycARTICLES and PsycINFO were searched, using the search terms (‘alopecia areata’) AND (‘adjust*’ OR ‘coping’ OR ‘*living*’ OR ‘experience’ OR ‘*psycholog*’ OR ‘*wellbeing’). A forward citation search was also conducted from key papers.^11 26 27^

Qualitative, quantitative and mixed-methods research published in English between 2003 and 2023 were included as articles used to inform deductive codes for the analysis of survey 1. The first author screened the 367 identified articles by title and reviewed the most relevant articles (43 articles) by abstract. They then read the full reports of the most relevant (13 articles) and searched for themes across the studies that could be used as deductive codes. Table 1 shows the articles and their corresponding codes used for analysis.

**Table 1:** Relevant articles from the literature review and their corresponding deductive codes

Authors	Methodology	Codes drawn from articles and used in the initial codebook
Aldhouse *et al*^32^	Qualitative interviews	Acknowledge complexity in wearing wigs and social interactions; recognise men can also struggle with AA; validate sense of loss and trauma
Barkauskaite and Serapinas^33^	Qualitative interviews	Nurture self-acceptance; validate the sense of loss and trauma; acknowledge the impact on perceived femininity
Cartwright *et al*^12^	Quantitative correlational survey	Attribute the cause of AA to chance; focus on emotional response to diagnosis over physical symptoms; reduce avoidant coping
Davey *et al*^11^	Qualitative survey	Validate enacted stigma; recognise the importance of hair to identity; explore and adapt an individual’s meaning attributed to hair; validate the sense of loss and trauma; peer support—attunement from others; peer support—validation; peer support—normalisation; early psychological intervention; nurture self-acceptance
Huck *et al*^34^	Quantitative correlational survey	Peer support—attunement from others; peer support—validation; peer support—normalisation
Hunt and McHale^26^	Qualitative survey and correspondence	Recognise the importance of hair to identity
Iliffe and Thompson^19^	Qualitative interviews	Peer support—attunement from others; peer support—validation; peer support—normalisation; nurture self-acceptance; active coping
Matzer *et al*^27^	Mixed-methods survey	Active coping; encourage perspective-taking; reduce avoidant coping; nurture self-acceptance; peer support—attunement from others; peer support—validation; peer support—normalisation
Montgomery *et al*^20^	Mixed-methods survey	Acknowledge the complexity of wearing wigs and social interactions; support with disclosure decisions
Welsh and Guy^35^	Qualitative interviews	Encourage perspective-taking; nurture self-acceptance; active coping
Wiggins *et al*^36^	Qualitative interviews, focus groups and video diaries	Acknowledge the complexity of wearing wigs and social interactions; support with disclosure decisions
Willemse *et al*^37^	Quantitative correlational survey	Attribute the cause of AA to chance; focus on emotional response to diagnosis over physical symptoms; reduce avoidant coping
Zucchelli *et al*^22^	Qualitative interviews	Acknowledge the impact on perceived masculinity; recognise men can also struggle with AA.

AA, alopecia areata.

### Delphi round 1: qualitative survey

#### Recruitment of panellists

Experts by support role were recruited by the first author via two primary methods: (1) professional contacts and networks and (2) in-person contact at an Alopecia UK national event in March 2023. Experts by experience were recruited at the event and via online advertising disseminated by Alopecia UK.

Inclusion criteria for experts by support role included being based in the UK, having at least 1 year’s experience of supporting individuals with AA and having provided psychological support to these individuals. For the study’s purposes, psychological support was defined as any talk-based psychological support provided via health organisations (including charities) or individuals in a formal support capacity. This includes support groups, talking therapies, health consultations (eg, with a GP or dermatologist) and cosmetic professional consultations (eg, trichologists providing wigs in dual capacities).

Inclusion criteria for experts by experience comprised experience of having received psychological support (as defined above) for challenges related to AA, being aged 18 years or above, being UK-based and being able to speak conversational English. Currently experiencing levels of distress that impair daily functioning was an exclusion criterion. Panellists could participate with dual roles, such as expert by experience and peer supporter.

The target sample size was 50, as recommended to gain sufficiently representative data for Delphi studies.^28^ In total, 68 individuals expressed interest in participating as a panellist and were invited by the first author to participate. Five responded but were ineligible for inclusion (eg, had never received psychological support or had not supported individuals with AA). 48 individuals completed the survey, representing a 76% (48/63) response rate. A breakdown by panellist role and demographic data for the group is shown in table 2. Three panellists held a dual role as experts by experience and support role (as peer support facilitators).

**Table 2:** Panellist information

	Round 1	Round 2	Round 3
All experts, N	48	46	23
Experts by experience			
n*	29	27	
Gender			
Male	3	3	
Female	26	24	
Age (mean, range)	43 (21–72)	43 (21–72)	
Ethnicity			
White British	27	25	
White other	0	0	
Black British	0	0	
Asian/Asian British	2	2	
Experts by support role			
n*	22	21	23
Support role			
Medical professionals	9	10	10
Mental health professionals	6	4	6
Trichologists	3	3	3
Peer supporters	4	4	4
Gender			
Male	4	3	4
Female	18	18	19
Age (mean, range)	39 (32–70)	41 (32–70)	40 (32–70)
Ethnicity			
White British	15	15	14
White other	1	1	2
Black British	2	1	2
Asian /Asian British	2	2	3
Chinese	1	1	1
Arabic	1	1	1

* The sum of n for each survey is greater than N because panellists with dual roles are counted within each role.

n, number of experts in subgroup; N, total number of experts.

#### Data collection

An online qualitative survey was informed by the research question, study advisor input and the literature review. The survey was delivered online via Qualtrics. Panellists were offered a £10 online shopping voucher for this and all subsequent rounds completed.

In the survey, panellists were directed to participant information followed by informed consent. Open-ended questions asked panellists for their insights on what they deemed as crucial aspects of psychological support for individuals with AA. Questions varied by role. Where panellists held dual roles, they were asked all questions relevant to both roles.

Examples of questions posed to experts by experience include ‘What aspects of the psychological support did you find helpful, and why?’ and ‘Is there any important practical advice you found helpful, or would have found helpful, to manage the appearance-altering element of your alopecia?’ Examples of questions for experts by support role were ‘Which aspects of peer support seem to be most helpful for people with alopecia to improve their quality of lives?’ (for peer supporters) and ‘In your consultations with people who have AA, are there any key messages or qualities of communication that you find help people with AA to live their life?’ (for medical professionals and trichologists). Please see the full surveys for rounds 1–3 in online supplemental file 2.

#### Data analysis

The first author analysed panellists’ data collectively using qualitative content analysis aimed at generating codes that could translate into actionable recommendations. They adopted an unconstrained codebook approach,^29^ in which deductive codes drawn from the literature review were used as an initial framework for coding data (shown in table 1). After familiarising themselves with the data and conducting open coding, additional, data-driven codes to describe recurring patterns in the data were created alongside the deductive codes to form an unconstrained codebook. The first author then categorised the initial codebook into categories and candidate recommendation items (subcategories) through a process of grouping and abstraction. This also included removing deductive codes where no relevant instances were noted in the dataset or merging them with inductive codes. The second author reviewed the initial codebook for coherence and comprehensiveness. The first author then coded all data for frequencies of items across the dataset. They completed this process iteratively, working through five versions of the codebook, culminating in a final codebook and set of frequencies.

## Results

Table 3 shows the candidate recommendation items generated through the content analysis, their overarching categories and the frequencies and percentages of panellists who described each item. The wording and structure of the version shown in table 3 incorporate changes informed by study advisors after reviewing its content. For example, the group suggested reorganising the categories to create a separate category on supporting children and young people. The online supplemental table 1 shows a full description of each item, including quotes from panellists.

**Table 3:** Candidate recommendation items from round 1 analysis with results from rounds 1 and 2

Category	Recommendation item	Round 1 (N=48)		Round 2 (N=46)		
		n and percentage of panellists describing item		Mean rating (1–5)	Percentage of panellists rating item moderately or very important	Mean ranking in category
Affected individuals’ perception of AA: its causes, outcomes and impact	Tactfully manage individuals’ expectations of AA outcomes	26	54%	4.78	100%	3.43
	Help individuals prepare for different possible AA outcomes and tolerate uncertainty	8	17%	4.85	100%	3.09
	Help individuals form a clear understanding of AA and make informed decisions	24	50%	4.48	100%	2.02
	Focus individuals away from self-blaming attributions of AA cause	4	8%	4.98	96%	4.24
	Validate (and explore) the emotional impact of AA	40	83%	4.78	100%	2.22
Supporting psychological and social adjustment to life with AA	Nurture individuals’ capacity to create positives from life with AA and take helpful perspectives	18	38%	4.52	96%	3.80
	Help individuals prepare for unwanted attention on their appearance	24	50%	4.54	98%	3.17
	Nurture individuals’ acceptance of themselves and the condition	11	23%	4.70	98%	1.83
	Help individuals to adopt authentic strategies to improve quality of life	16	33%	4.48	96%	3.30
	Encourage physical self-care	6	13%	4.24	89%	4.65
	Support individuals to make decisions about telling/showing others about their AA and how they can do this	12	25%	4.43	91%	4.24
Awareness from the supporter	Respect and work with individuals’ chosen coping strategies (where no clear harm is caused)	18	38%	4.73	98%	3.49
	Be aware of any unhelpful coping strategies individuals are using	5	10%	4.82	100%	3.42
	Allow for time and fluctuation within individuals while adjusting to life with AA	19	40%	4.64	96%	3.23
	Hold in mind that individuals’ identities may shape their experiences	16	33%	4.73	98%	3.60
	Recognise variation between individuals in responding to a diagnosis of AA	11	23%	4.78	100%	2.35
	Cultivate self-awareness of your own assumptions and attitudes about appearance	12	25%	4.64	100%	4.91
Delivering support	Psychological support should be accessible and flexible to individuals’ needs and preferences	35	73%	4.73	96%	3.14
	(Formal) psychological support should be offered early on from diagnosis	21	44%	4.89	100%	1.79
	There should be a holistic, multi-support-role and multisector approach to psychological support	21	44%	4.51	89%	2.74
	Give time in-session or acknowledge time constraints for individuals to share their experiences	12	25%	4.60	98%	3.44
	Peer supporters need to skilfully manage the complexities of peer groups	11	23%	4.67	100%	3.88
Supporting children and young people	Consider children’s and young people’s context	13	27%	4.88	100%	2.06
	Communicate sensitively with children and young people	6	13%	4.83	100%	1.83
	Involve and support the families of children and young people with AA	6	13%	4.88	100%	2.11

AA, alopecia areata; N, total number of experts; n, number of experts in subgroup.

### Delphi round 2: rating survey

#### Recruitment of panellists

Recruitment for study 2 was conducted in a staggered fashion. First, all 48 round 1 panellists were invited to participate. After 2 weeks (by which point 35 round 1 panellists had completed round 2), the first author also invited all 15 remaining eligible individuals who had originally expressed interest in the study. In total, 46 panellists took part: 42 of whom had completed round 1 and 4 of whom had not. The response rate was 73% (46/63). Full information for round 2 panellists is presented in table 2.

#### Data collection

In the rating survey, panellists were presented with each recommendation item as an actionable statement and a brief applied definition (including quotes from panellists’ round 1 responses). The item statements and categories are shown in table 3. Online supplemental table 1 shows an example item with a definition.

Panellists were asked to rate the importance of each item on a scale of 1–5 (1*=not at all important,* 5*=very important*). Experts by support role were advised to answer from the perspective of a member of the wider support team, meaning they may rate a recommendation directed more towards those in other support roles as more important than one directed more towards their role. Experts by experience were advised to rate from the point of view of an individual seeking the best possible psychological support. Panellists were also asked to rank the importance of the items within their categories to further discern between items’ perceived importance. Panellists were also invited to provide optional qualitative feedback on each item statement to suggest changes to the item summaries and/or their definitions.

#### Data analysis

Rating data were analysed descriptively via frequencies and percentages of rating scores and mean ratings. Consensus was determined via a commonly used threshold of ≥80% agreement across panellists that items were either *moderately* (4) or *very important* (5) and a mean importance score of ≥4.^30^ For comprehensiveness, items were to be removed from the recommendations if they were ranked as least important on average within their category and more panellists rated them as moderately than very important. Qualitative feedback was not analysed systematically due to the low number of responses for each item but instead treated as stakeholder input to inform round 3.

#### Results

Mean ratings, frequencies and percentages and mean rankings are shown by item in table 3. All items met the consensus threshold other than ‘Encourage physical self-care’, which was rated as *moderately important* by more panellists than *very important* (52% vs 37%) and was ranked lowest within its category on average. The items ranked as most important within their categories on average were ‘Validate (and explore) the emotional impact of AA’, ‘Recognise variation between individuals in responding to a diagnosis of AA’, ‘(Formal) psychological support should be offered early on from diagnosis’ and ‘Communicate sensitively with children and young people’.

### Round 3: modified Delphi round

#### Change to study protocol

Rather than proceeding with a re-rating survey in round 3 per protocol, the authors and study advisors decided to reorient round 3 towards developing role-specific recommendations. This decision was made for two reasons.

First, consensus was achieved in round 2 for all but one item. While re-rating items gives panellists the opportunity to reconsider their ratings in line with group norms, the additional task demand on panellists is less justifiable when a clear consensus is already reached, especially where there is no defined constraint to the number of final items.^23^

Second, panellist feedback in round 2 indicated that item wording and relevance will vary greatly by specific roles. For example, medical professionals may be better placed to ‘Help individuals form a clear understanding of AA and make informed decisions’, while mental health professionals may have more dedicated time and skills to ‘Help individuals to adopt authentic strategies to improve quality of life’.

#### Recruitment of panellists

To maximise sample size, 27 experts by support role were invited to participate: 21 of whom had completed round 2, 3 of whom had completed round 1 and 3 of whom had expressed interest but not yet participated. 23 completed the survey, a response rate of 85% (23/27). Table 2 shows a breakdown by role and demographic data for panellists.

#### Data collection

In the online survey, panellists were first presented with anonymised summary information from round 2, including the rankings for recommendations in each category, a summary of the frequencies and examples of reworded recommendations based on their round 2 feedback. Panellists were then asked to categorise the relevance of each remaining item as *Not*, *Partly* or *Directly…relevant to/suitable for my role*. They were also invited to explain their choices and suggest whether there are any modified versions of the items that would be more relevant/suitable.

#### Data analysis

Frequencies of categories were recorded, and items categorised as *directly* relevant by more than half of panellists within each role were automatically included in the corresponding final recommendations. Where under half of panellists within each role had categorised them as *directly* relevant but had rated them as *partly* relevant and provided suggestions of how they could be made more relevant, the first author reviewed these on a case-by-case basis and reworded items and their definitions where deemed appropriate.

#### Results

Table 4 shows which items were retained in each set of recommendations by support role, using the above criteria. Items retained in all four versions were ‘Validate (and explore) the emotional impact of AA’, ‘Respect and work with individuals’ chosen coping strategies (where no clear harm is caused)’ and ‘There should be a holistic, multi-support-role and multisector approach to psychological support’.

**Table 4:** Recommendation items by support role following Round 3

Category	Recommendation item	Retained in support role-specific recommendations			
		Medical professional	Mental health professional	Peer support	Trichology
Affected individuals’ perception of AA: its causes, outcomes and impact	Tactfully manage individuals’ expectations of AA outcomes	x			x
	Help individuals prepare for different possible AA outcomes and tolerate uncertainty		x	x	
	Help individuals form a clear understanding of AA and make informed decisions	x		x	x
	Be sensitive to how individuals may perceive suggestions of stress-based causes of AA	x	x		x
	Validate (and explore) the emotional impact of AA	x	x	x	x
Supporting psychological and social adjustment to life with AA	Nurture individuals’ capacity to create positives from life with AA and take helpful perspectives		x	x	
	Nurture individuals’ acceptance of themselves and the condition	x	x	x	
	Help individuals prepare for unwanted attention on their appearance		x		
	Help individuals to adopt authentic strategies to improve quality of life		x		
	Support individuals to make decisions about telling/showing others about their AA and how they can do this		x	x	
Awareness from the supporter	Respect and work with individuals’ chosen coping strategies (where no clear harm is caused)	x	x	x	x
	Be aware of any unhelpful coping strategies individuals are using	x	x	x	
	Allow for time and fluctuation within individuals while adjusting to life with AA	x	x		
	Recognise variation between individuals in responding to a diagnosis of AA	x	x	x	
	Hold in mind that individuals’ identities may shape their experiences	x	x	x	
	Cultivate self-awareness of your own assumptions and attitudes about appearance		x	x	
Delivering support	Psychological support should be accessible and flexible to individuals’ needs and preferences		x	x	
	Refer, signpost to or offer psychological support early on from diagnosis	x		x	x
	There should be a holistic, multi-support-role and multisector approach to psychological support	x	x	x	x
	Give time in session or acknowledge time constraints for individuals to share their experiences		x		
	Peer supporters need to skilfully manage the complexities of peer groups			x	
Supporting children and young people	Consider children’s and young people’s context	x	x		x
	Communicate sensitively with children and young people	x	x		x
	Involve and support the families of children and young people with AA	x	x		

AA, alopecia areata.

Following panellists’ feedback, multiple items were reworded for different support roles. Examples of reworded items included: removing ‘(and) explore’ from ‘Validate and explore the emotional impact of AA’ for medical professionals, from their feedback that this was realistically beyond their time and skill resources. Where items were reworded for all versions of recommendations, the new wording is shown in table 4.

From study advisor and peer panellist feedback, the item ‘Peer supporters need to skilfully manage the complexities of peer groups’ was divided into smaller, more actionable items derived from the definition of the item from round 1. The new items include ‘Recognise the range in peer group members’ level of AA progression and adjustment’, ‘Sensitively set boundaries around sharing traumatic experiences’, ‘Be aware of imbalances in age, gender and ethnicity’ and ‘Make the environment a ‘safe space’’. The first author sent all final recommendations to the relevant round 3 panellists to review, with no further changes requested.

## Discussion

In this study, we used a modified Delphi study to systematically develop recommendations for care providers to promote well-being in individuals with AA. Informed by panellist and study advisory feedback, tailored recommendations by care provider role were developed for greater specificity and relevance.

Findings across the three rounds indicate that validation and exploration of the emotional impact that AA can exert on individuals is *the* primary recommendation for care providers. From panellists’ own views on the key aspects of wellbeing promotion in round 1, over 80% described this need; it was ranked as most important within its category in round 2, and retained within all four recommendation versions in round 3. Validation of AA’s potential impact by medical professionals, with whom individuals consult before any other care provider, may be particularly important. This follows research suggesting that empathy from medical professionals, and especially greater awareness about the disruption to self-identity and social interactions, is desired by individuals with AA.^13^

Findings also point to helpful demarcation in roles and perceived responsibilities by care providers in managing affected individuals’ wellbeing. Over half of panellists described managing expectations of AA outcomes as a key factor in promoting well-being in round 1. Its retention in recommendations for medical professionals and trichologists from round 3 supports the idea that this is a vital part of their role, with AA outcomes often unpredictable.^3^ Amid a shifting landscape of treatment effectiveness and availability,^31^ in which affected individuals’ expectations may be higher than ever for hair regrowth, this responsibility is greater than ever for treating professionals.

Of note, mental health professionals and peer supporters did not deem managing expectations as relevant to their role, but they deem the recommendation of helping individuals to prepare for different outcomes and tolerate uncertainty as highly relevant . This indicates a clear division of expertise and responsibilities, in which medical professionals can convey possible hair regrowth outcomes, mental health professionals support individuals to process this information, and peers facilitate the sharing of practical means of managing appearance changes (eg, concealment strategies like wigs, headwear and medical tattooing). All care providers working in a joined-up, multidisciplinary, multisector manner would clearly enable this process, which is backed by this recommendation being retained in all four role-specific recommendations.

A strength of the study was its pragmatic orientation towards developing actionable recommendations for the multidisciplinary, multisector care providers, recognising the responsibility of all involved to promote well-being in the individuals they support. Previous research has explored coping strategies in individuals with AA^11 27^, and although this has provided some direction towards aspects of well-being promotion generated in the present study (eg, self-acceptance), other identified coping strategies may be less amenable to care provider intervention (eg, social support from significant others). Other studies have investigated the efficacy of specialist-led psychological interventions,^14 15^ which are less applicable across multiple care provider roles. Nevertheless, further research developing and testing PPI-informed psychological interventions for individuals with AA remains paramount.

A further strength of the study was the inclusion of experts by experience in developing the recommendations; an often-overlooked group when creating clinical recommendations or guidelines.^24^ A responsive, flexible methodological approach also incorporated panellist and study advisor input throughout, meaning stakeholders’ preferences guided the outcome towards tailored recommendations.

There was an under-representation from Black and Asian individuals, most notably in the expert by experience group. This is particularly significant given that recent UK research highlights a 1.5-fold and threefold incidence of AA diagnosis in Black and Asian individuals, respectively.^2^ While the role of identity and culture was covered in the recommendations, we may have missed important cultural, racialised and ethnic differences due to this under-representation. There remains a great need for research to better understand the support needs of Black and Asian individuals with AA.

Men were also underrepresented in the panellist group. This was at least partly mitigated through the inclusion of research on men’s experiences of AA in the analysis of study 1, and no relevant items were subsequently removed during the rating rounds. Another group for whom the recommendations may provide limited detail is children and young people. From round 1, it was apparent that some experts by experience had developed AA in childhood and offered views on supporting this age group. However, more age-specific research is required to more comprehensively understand and address the support needs of children and young people.

It is also important to recognise the potential influence of the expert by experience group of factors, like age, time since AA onset and type of well-being support received on their perspectives, on optimal well-being promotion. The study design precluded closer examination of these factors. In the expert by support role group, there were a higher number of medical professionals than in other roles, potentially leading to greater emphasis on recommendations more suited to this role. However, round 3 involving a role-specific rating of relevance did protect against this.

By using systematic yet responsively malleable consensus methods, this study offers the most practical direction to date for care providers involved in supporting individuals with AA. Dissemination to care providers is now key to delivering consistency across the roles while also complementing the respective skill sets and remits of each role to promote well-being in individuals with AA. This can be achieved by using the recommendations as a basis for educational resources across the different professional organisations (eg, British Association for Counselling and Psychotherapy; Primary Care Dermatology Society) and incorporating the recommendations into existing professional guidelines (eg, British Association of Dermatologists’ living guidelines for managing AA).

## Supplementary material

10.1136/bmjopen-2024-094491 online supplemental file 1

10.1136/bmjopen-2024-094491 online supplemental file 2

## Data Availability

Data are available upon reasonable request.
