# Supplementary material for: Developing recommendations for promoting wellbeing in individuals with alopecia areata: a modified Delphi study
Source: BMJ Open. 2026 Feb 10;16(2):e094491. doi: 10.1136/bmjopen-2024-094491 (PMC12911720; doi:10.1136/bmjopen-2024-094491)
Supplement: online supplemental file 1 [file bmjopen-16-2-s001.docx]

**Supplementary Table 1. All recommendation items following survey 1, including quotes from panellists.**

| *Category* | *Recommendation item* | *Full description of item, including quotes from panellists* |
| --- | --- | --- |
| Affected individuals' perception of AA: It's causes, outcomes and impact | Tactfully manage individuals' expectations of AA outcomes | Health professionals being realistic, open & honest about (1) prognosis (long-term outcome), (2) wider state of knowledge about AA, & (3) referral routes & wait times. Not giving "false hope", also "striking the right balance of being realistic but not pessimistic", aware of how affected individuals can unhelpfully get "caught up in the cycle of hope". Communicating this sensitively. |
|  | Help individuals prepare for different possible AA outcomes & tolerate uncertainty | Collaborate with individuals to talk through different possible AA outcomes & preparing for these. May involve (1) emotional preparation (e.g. "allow space to tolerate uncertainty"; "how they will cope if [hair loss] returns in the future") & (2) practical preparation (e.g. "having a think about wigs etc") |
|  | Help individuals form a clear understanding of AA & make informed decisions | Help individuals form coherent understanding of AA, via (1) "possible causes" (e.g. "considering other auto-immune conditions that may need to be addressed"), (2) treatment options (e.g. "various treatments we could try"), including "possible benefits" and side-effects, (3) camouflage options (e.g. "discuss options like scalp micropigmentation, brow microblading, wigs... etc so they feel empowered to make informed decisions", & (4) research opportunities (e.g. "made me aware of ongoing trials etc.") |
|  | Focus individuals away from self-blaming attributions of AA cause | Aware of potential for self-blame, regret & frustration in individuals when information & discussion focus on stress-based causes of AA (e.g. "[When] told that stress is the cause of hair loss, [some] try to identify this or start questioning their personality type"). Note that many people experience stress but don’t develop AA. Focus on autoimmune aspects and/or chance causes & support individuals to make sense of their AA. |
|  | Validate (and explore) the emotional impact of AA | Taking individuals' emotional difficulties seriously via active listening, "asking open questions about how their alopecia impacts them", empathy & nonjudgement. "Give permission..." that "...all feelings are valid". Aware of common feelings of grief "of my hair", loss of identity, trauma, isolation, sense of difference, & guilt (e.g. "its not cancer so why am I so upset about hair"). Acknowledge impact of dismissive responses from others (e.g. "because it's not life threatening, friends can be somewhat dismissive about it"). Aware that "prior experiences of loss, hopelessness, invalidation can all be reactivated by alopecia". |
| Supporting psychological and social adjustment to life with AA | Nurture individuals' capacity to create positives from life with AA and take helpful perspectives | Help individuals focus on positives parts of self "aside from just appearance" (e.g. to "realise that my hair didn't define me"). Or help "focus on other things about your appearance - make-up, clothes, fitness so you feel good about the rest of your body". Help take healthy perspectives, e.g. where appropriate "looking positively at your own good health". Recognise personal growth, e.g. "many [want to] help others and this also keeps some coming [to peer events]". Broach gently & at individuals' pace, as otherwise can feel dismissive of current impact. |
|  | Nurture individuals' acceptance of themselves & the condition | Help individuals grow - & recognise importance (& challenge) of – (1) self-acceptance (e.g. "[discussing] how they can fall back in love with themselves whilst adjusting to the new reflection in the mirror”) & (2) acceptance of AA (e.g. "acceptance about the [lack of reliable] cure"). Help individuals "own the alopecia", by integrating their new appearance into their identity, whether through products/clothing (e.g. headwear) or "embracing bald". |
|  | Help individuals prepare for unwanted attention on their appearance | Acknowledge stigma towards different appearance "in a society that places value on appearance". Help individuals "manage reactions of others - having prepared responses", "discuss ways to deal with certain situations e.g. questions about alopecia - a brief explanation they can learn, then change the subject, etc.". "Self-care after [interactions] they know will be challenging." |
|  | Help individuals to adopt authentic strategies to improve quality of life | Support individuals to adopt emotional, physical and practical coping strategies that feel authentic: Consider their (1) personality & values (e.g. "making light of my alopecia... that suited my personality, it wouldn't suit everyone") & (2) their valued activities (e.g. "as an extremely active individual, the practicalities of wearing a wig were not complimentary to my lifestyle... so I [chose to wear] a headband"). |
|  | Encourage physical self-care | Recognise physical self-care as part of healthy adjustment to condition, & its overall health & wellbeing benefits. Could include (1) "skin and scalp care", (2) exercise & fitness, (3) diet, & (4) "adjustments for [physical] comfort… e.g. type of pillow". |
|  | Support individuals to make decisions about telling/showing others about their AA & how they can do this | Help individuals decide whether, how & when to share AA diagnosis with others.. Enabling them to do so on their terms. Might involve actively (1) telling selected people (e.g. "open[ed] up to my family and close friends as well as work, once I did it was no longer a secret that I felt ashamed of..." or (2) 'broadcasting' it (e.g. "when you [feel ready], just tell everyone in one go; social media is a good way - like pulling off a plaster fast." Recognise challenges like "having to explain it to new people", & anxiety around wearing wigs & accidentally revealing AA. |
| Awareness from the supporter | Respect and work with individuals' chosen coping strategies (where no clear harm is caused) | Recognise "telling someone they don't need to wear a wig or [they] shouldn't hide from people when they are not ready can be very unhelpful and actually very distressing", & "I don’t like being in a forum... where there is pressure to go bald - You need to manage AA in your own way." Supporting individuals’ chosen practical & emotional coping strategies (e.g. wearing wigs, or focused on hair regrowth). Focus on whether their strategies aid or hinder quality of life, e.g. if wearing a wig feels necessary to leave house, it may be aiding quality of life. |
|  | Be aware of any unhelpful coping strategies individuals are using | Aware of when individuals are adopting understandable yet potentially unhelpful coping strategies long-term; like (1) withdrawing from friends & family (2) denying problem (e.g. "subconsciously hiding it away and 'wish[ing] it away'"), or (3) avoiding valued activities or opportunities. Sensitively helping them become aware of any unhelpful consequences of their strategies. |
|  | Allow for time & fluctuation within individuals while adjusting to life with AA | Patience towards individuals who may need time to adjust to diagnosis (e.g. "my grief was deep and long before I could begin to "put myself back together""). Aware of "rollercoaster… [of] cycles of growth, loss, watching, checking, masking etc". Working at their pace of adjustment & readiness to receive support. Depending on supporter's role, could involve offering follow-up "review to check up on me" and/or ongoing support to provide "continuity of care". Keeping support options on agenda throughout timespan of consultations. |
|  | Recognise variation between individuals in responding to a diagnosis of AA | Recognise individuals respond differently to diagnosis. Aware that extent of hair loss doesn't necessarily predict level of distress ("for some people, a small bald patch is devastating, while others with more severe symptoms adapt quickly"). Aware that some individuals may mask their suffering ("people with AA are often just putting on a brave face - while feeling really shit!") |
|  | Hold in mind that individuals' identities may shape their experiences | Aware that individuals' identities & backgrounds can influence their experience of living with AA, & their support needs, e.g. ethnicity, race &/or cultural background (e.g. "[In] Asian [culture] a lot of importance is placed on your hair"), gender (e.g. "My hair and my eyelashes, as a woman... when I lost them , I lost my femininity" and "Males, particularly masculine gendered males, cannot wear wigs or eyelashes"), sexuality. |
|  | Cultivate self-awareness of your own assumptions & attitudes about appearance | "Good psychological support means [you] have worked with your own bias and beauty ideals, [so you] do not unknowingly influence or judge the client or their choices." Being aware that men with AA can also struggle to adjust (e.g. "[An unhelpful belief is that] men suffer less than women because society got used to male pattern baldness, and they can [all] accept it easily and make jokes about it. False.") Awareness that individuals don't necessarily want reassurance about their appearance (e.g. "I've been told many times that it's really irritating when people try to reassure"). |
| Delivering support | Psychological support should be accessible and flexible to individuals' needs & preferences | Support offered by mental health professionals or trained peers should be flexible to accommodate variation in individuals' preferences regarding: (1) Format (i.e. in-person or online; 1-to-1 or group) ,"[support should be] offered in different formats to suit different people", & (2) Setting, i.e. in healthcare settings ("[some] prefer to be seen in the hospital where they attend appointments") or community ("medical settings are too cold and daunting.") "Readily available" support where possible. |
|  | (Formal) psychological support should be offered early on from diagnosis | Though support should be on agenda throughout individuals' AA journey, it should always be made available from initial consultations. This could help speed up adjustment processes (e.g. "I wish I had had psychological and peer support earlier - I think this may have helped me to come to terms with my alopecia at an earlier stage") & provide vital coping strategies (e.g. "I do think some of the negative aspects of the condition wouldn't have had the chance to take hold... healthy coping techniques are essential!") |
|  | There should be a holistic, multi-support-role & multisector approach to psychological support | "The best service involves a holistic approach (i.e. addressing all health & wellbeing aspects) with all professionals working together". Multi-support-role involvement with communication, signposting & shared input & learning, across support roles (e.g. "[As a mental health professional] I find it very helpful to link in with my medical colleagues to help understand the medical context", & "I would like to have been signposted to [charitable] support by a health professional". Multisector approach, working across medical, charitable & private sectors to provide joined up care. |
|  | Give time in-session or acknowledge time constraints for individuals to share their experiences | Wherever possible, give "time [in-session] to express how the alopecia is making them feel e.g. loss of confidence, loss of identity etc". Given health professionals' time pressures especially in NHS settings, openly naming this challenge, & signposting to support sources (including peer support) where more time can be given. |
|  | Peer supporters need to skilfully manage the complexities of peer groups | Peer facilitators' awareness of potential challenges of peer groups, & ability to manage: (1) Range of members' AA progression and adjustment (e.g. "if someone is really struggling and meeting others who are really positive this can have the opposite effect of being unsupportive and invalidating"), (2) members sharing "horror stories" & high expressed emotion, (3) Gender/age/ethnicity imbalances (e.g. "it might not be helpful for a twelve year old to be at a peer support meeting with a group of 50 year old women", & (4) Cliques forming. Need to create safe space by welcoming new members, "giving people plenty of info beforehand so they know what they are coming too, and are prepared". |
| Supporting children & young people | Consider children & young people’s context | Aware of childhood & adolescence context: (1) Still-forming identity (e.g. "interrupt" development of "a strong sense of self") & centrality of feeling accepted by peers, (2) Milestones (e.g. "when I left school, started university, travelled etc... there are always aspects of having alopecia that will surprise you"), & (3) Potential for teasing &/or bullying. |
|  | Communicate sensitively with children & young people | Use age-appropriate language (e.g. "keeping literature and the way it is communicated to [children] fun and simple") & sensitivity to how overwhelming (or "terrifying”) consultations can be for children or young people |
|  | Involve and support the families of children & young people with AA | Recognise family members’ needs, "involving parents and other family members as appropriate" in supporting the affected child or young person. Providing "support [for] parents of children suffering with hair loss so they to give them the tools to support their child". Also gently address families’ attitudes (explicit or implied) that may impact on the child / young person: "Children [can] suffer from their parent's feelings about their baldness" (e.g. wanting their child to wear a wig and the potential shame this implies). |
