# Supplementary material for: Developing recommendations for promoting wellbeing in individuals with alopecia areata: a modified Delphi study
Source: BMJ Open. 2026 Feb 10;16(2):e094491. doi: 10.1136/bmjopen-2024-094491 (PMC12911720; doi:10.1136/bmjopen-2024-094491)
Supplement: online supplemental file 2 [file bmjopen-16-2-s002.docx]

Survey 1 - AA Delphi

Q2 In which capacity as an alopecia expert would you like to participate? *(You can choose more than one. Equally, even if you meet the criteria for more than one, you can choose to only answer in one capacity)*

- Expert by support role (1)
- Expert by lived experience (2)
- Expert by research (3)

| Page Break |  |
| --- | --- |

Start of Block: Experts by support role

Q10 Which best describes your support role?

- Mental health professional (e.g. psychologist, counsellor, psychotherapist, wellbeing practitioner) (1)
- Peer supporter (3)
- Health professional (e.g. dermatologist, GP, nurse) (2)
- Trichologist  (4)
- Cosmetic professional (e.g. wig or medical tattoo provider) (5)

| Page Break |  |
| --- | --- |

Q11 Please draw on your experience from providing psychological support for people who have alopecia (areata, totalis or universalis) when answering the following questions. You can answer all or only some of the questions. Please give as much detail as you feel able, as this will help provide rich information to analyse.

| Page Break |  |
| --- | --- |

Q12
What do you consider important to hold in mind when working with people who have alopecia?

________________________________________________________________

________________________________________________________________

________________________________________________________________

________________________________________________________________

________________________________________________________________

Display this question:

If Which best describes your support role? = Mental health professional (e.g. psychologist, counsellor, psychotherapist, wellbeing practitioner)

Q13 Which approaches and/or techniques have you found particularly helpful for supporting people with alopecia to improve their quality of life?

________________________________________________________________

________________________________________________________________

________________________________________________________________

________________________________________________________________

________________________________________________________________

Display this question:

If Which best describes your support role? = Peer supporter

Q14 Which aspects of peer support seem to be most helpful for people with alopecia to improve their quality of life?

________________________________________________________________

________________________________________________________________

________________________________________________________________

________________________________________________________________

________________________________________________________________

Display this question:

If Which best describes your support role? = Trichologist

Or Which best describes your support role? = Cosmetic professional (e.g. wig or medical tattoo provider)

Or Which best describes your support role? = Health professional (e.g. dermatologist, GP, nurse)

Q16 In your consultations, are there any key messages or qualities of communication that you find help people with alopecia to live their life?

________________________________________________________________

________________________________________________________________

________________________________________________________________

________________________________________________________________

________________________________________________________________

| Page Break |  |
| --- | --- |

Display this question:

If Which best describes your support role? = Mental health professional (e.g. psychologist, counsellor, psychotherapist, wellbeing practitioner)

Q17 If there are any approaches and/or techniques have you found to be unhelpful for supporting people with alopecia to improve their quality of life, what are they, and why are they unhelpful?

________________________________________________________________

________________________________________________________________

________________________________________________________________

________________________________________________________________

________________________________________________________________

Display this question:

If Which best describes your support role? = Peer supporter

Q18 If there are any aspects of peer support that seem to be unhelpful for people with alopecia to improve their quality of life, what are they, and why are they unhelpful?

________________________________________________________________

________________________________________________________________

________________________________________________________________

________________________________________________________________

________________________________________________________________

Display this question:

If Which best describes your support role? = Trichologist

Or Which best describes your support role? = Cosmetic professional (e.g. wig or medical tattoo provider)

Or Which best describes your support role? = Health professional (e.g. dermatologist, GP, nurse)

Q19 In your consultations, are there any messages or qualities of communication that you find unhelpful for people with alopecia?

________________________________________________________________

________________________________________________________________

________________________________________________________________

________________________________________________________________

________________________________________________________________

Display this question:

If Which best describes your support role? = Mental health professional (e.g. psychologist, counsellor, psychotherapist, wellbeing practitioner)

Q20 Are there any approaches and/or techniques you have found particularly helpful to engage and/or keep people with alopecia engaged in support?

________________________________________________________________

________________________________________________________________

________________________________________________________________

________________________________________________________________

________________________________________________________________

Display this question:

If Which best describes your support role? = Peer supporter

Q21 Are there any things that seem to help people with alopecia keep engaged with peer support?

________________________________________________________________

________________________________________________________________

________________________________________________________________

________________________________________________________________

________________________________________________________________

| Page Break |  |
| --- | --- |

Display this question:

If Which best describes your support role? != Cosmetic professional (e.g. wig or medical tattoo provider)

Q22 Are there any tips or approaches you have found helpful to support people around the progression of their alopecia and/or treatment?

________________________________________________________________

________________________________________________________________

________________________________________________________________

________________________________________________________________

________________________________________________________________

Q23 Are there any tips or approaches you have found helpful when supporting people to manage the appearance-altering element of alopecia? *(e.g. in social or intimate interactions, with or without wigs)*

________________________________________________________________

________________________________________________________________

________________________________________________________________

________________________________________________________________

________________________________________________________________

| Page Break |  |
| --- | --- |

Display this question:

If Which best describes your support role? != Cosmetic professional (e.g. wig or medical tattoo provider)

Q24 What might the ideal support service for people with alopecia look like? *e.g. in terms of:*  *format(s) – i.e. in person, online etc.* *timing in relation to diagnosis/progression* *place within health or other services*

________________________________________________________________

________________________________________________________________

________________________________________________________________

________________________________________________________________

________________________________________________________________

Q25 Do you want to share anything else that addresses the question: “What does good psychological support for alopecia look like?”

________________________________________________________________

________________________________________________________________

________________________________________________________________

________________________________________________________________

________________________________________________________________

End of Block: Experts by support role

Start of Block: Experts by lived experience

Q27 Please draw on your personal experience of receiving psychological support when answering the following questions. You may wish to focus on one particular form of psychological support you received, or more. You can answer all or only some of the questions. Please give as much detail as you feel able, as this will help provide rich information to analyse.

| Page Break |  |
| --- | --- |

Q26
What aspects of the psychological support did you find helpful, and why?

________________________________________________________________

________________________________________________________________

________________________________________________________________

________________________________________________________________

________________________________________________________________

Q28

​​​​​​If there were any aspects that you found less helpful, what were they, and how could they have been improved?

________________________________________________________________

________________________________________________________________

________________________________________________________________

________________________________________________________________

________________________________________________________________

| Page Break |  |
| --- | --- |

Q30 If you received support more than once, and at different stages along your alopecia journey, how did they compare?

________________________________________________________________

________________________________________________________________

________________________________________________________________

________________________________________________________________

________________________________________________________________

Q29


Do you have any thoughts on the timing of the psychological support you received, and how this might have impacted your experience? *​​​​​​​By timing, this could be in relation to your diagnosis, hair loss progression, or age at the time.*

________________________________________________________________

________________________________________________________________

________________________________________________________________

________________________________________________________________

________________________________________________________________

| Page Break |  |
| --- | --- |

Q31
​​​​​​In what kind of setting would you ideally like to have accessed psychological support? *(e.g. in person or online; one-to-one or group; medical or community settings)* *You can also share any relevant experiences of receiving support in particular settings, and whether you were given a choice.*

________________________________________________________________

________________________________________________________________

________________________________________________________________

________________________________________________________________

________________________________________________________________

Q32

​​​​​​Is there anything you think people should hold in mind when supporting someone who has alopecia?

________________________________________________________________

________________________________________________________________

________________________________________________________________

________________________________________________________________

________________________________________________________________

| Page Break |  |
| --- | --- |

Q33


​​​​​​Are there any helpful ways of coping with your alopecia that you have developed yourself, which could be nurtured through psychological support?

________________________________________________________________

________________________________________________________________

________________________________________________________________

________________________________________________________________

________________________________________________________________

Q34


​​​​​​Is there any important practical advice you were given, or wish you'd been given, about managing treatment of your alopecia?

________________________________________________________________

________________________________________________________________

________________________________________________________________

________________________________________________________________

________________________________________________________________

| Page Break |  |
| --- | --- |

Q35


​​​​​​Were there any helpful messages you were given by any health professionals, or wish you'd been given, about how your alopecia would progress over time (the "prognosis")?

________________________________________________________________

________________________________________________________________

________________________________________________________________

________________________________________________________________

________________________________________________________________

Q36


Is there any important practical advice you found helpful, or would have found helpful, to manage the appearance effects of your alopecia? *(e.g. in social or intimate interactions, with or without wigs)*

________________________________________________________________

________________________________________________________________

________________________________________________________________

________________________________________________________________

________________________________________________________________

| Page Break |  |
| --- | --- |

Q37 Do you want to share anything else that addresses the question: “What does good psychological support for alopecia look like?”

________________________________________________________________

________________________________________________________________

________________________________________________________________

________________________________________________________________

________________________________________________________________

Q51 Thank you very much for answering these questions, which I appreciate may have brought up difficult memories and feelings. It's only because people like you are willing to share their experiences, that we can create meaningful recommendations for psychological support for alopecia.

End of Block: Experts by lived experience

Start of Block: Experts by research

Q39 Please draw on your scientific expertise when considering the following questions. You may choose to answer all or only some of the questions. Please give as much detail as you feel able, as this will help provide rich information to analyse.

| Page Break |  |
| --- | --- |

Q40
Are there any common experiences or concerns in individuals living with alopecia that need addressing and/or acknowledgement by those providing psychological support?

________________________________________________________________

________________________________________________________________

________________________________________________________________

________________________________________________________________

________________________________________________________________

Q41
​​​​​​Which coping strategies, if any, are indicated as worthwhile targets for psychological support for people living with alopecia? 
 *(This could be nurturing helpful strategies and/or reducing unhelpful ones)*

________________________________________________________________

________________________________________________________________

________________________________________________________________

________________________________________________________________

________________________________________________________________

| Page Break |  |
| --- | --- |

Q42

Are there any modifiable beliefs (e.g. about alopecia) that should be targeted through psychological support for people with alopecia?

________________________________________________________________

________________________________________________________________

________________________________________________________________

________________________________________________________________

________________________________________________________________

Q45 Are there any approaches and/or techniques that show promise or are indicated for supporting people with alopecia to improve their quality of life?

________________________________________________________________

________________________________________________________________

________________________________________________________________

________________________________________________________________

________________________________________________________________

| Page Break |  |
| --- | --- |

Q46 Which approaches are indicated to help people manage the appearance-altering element of alopecia? *(e.g. in social or intimate interactions, with or without wigs)*

________________________________________________________________

________________________________________________________________

________________________________________________________________

________________________________________________________________

________________________________________________________________

Q48 What might the ideal support service for people with alopecia look like? *e.g. in terms of:*  *format(s) – i.e. in person, online etc.* *timing in relation to diagnosis/progression* *place within health or other services*

________________________________________________________________

________________________________________________________________

________________________________________________________________

________________________________________________________________

________________________________________________________________

| Page Break |  |
| --- | --- |

Q49 Do you want to share anything else that addresses the question: “What does good psychological support for alopecia look like?”

________________________________________________________________

________________________________________________________________

________________________________________________________________

________________________________________________________________

________________________________________________________________

End of Block: Experts by research

AA Delphi Survey 2

Start of Block: Theme 1

Q102 In this survey you'll see a series of recommendations for you to rate and rank in terms of their importance to you. They will be grouped by theme. For each recommendation, there will be a 'headline' summary alongside its definition. The definitions include some quotes from survey 1. You'll also be invited to suggest changes to the definitions.

_______________________________ **IMPORTANT**  Some recommendations will apply to some support roles more than others - e.g. the more in-depth psychological work will apply more to mental health professionals, medically-informed support more to health professionals, and peer support more to the peer support facilitators. So please answer from the point of view of either: A member of the wider support team (if an expert by support role)

Someone wanting to (have) receive(d) the best psychological support (if an expert by lived experience) This means for experts by support role, you could still consider a recommendation directed more towards those in other support roles as more important than one directed more towards your role. ________________________________

| Page Break |  |
| --- | --- |

Q6 **Tactfully manage individuals' expectations of AA outcomes** - Health professionals being realistic, open & honest about: (1) Prognosis (long-term outcome) (2) Wider state of knowledge about AA (3) Referral routes & wait times. - Not giving "false hope", also "striking the right balance of being realistic but not pessimistic", aware of how affected individuals can unhelpfully get "caught up in the cycle of hope". Communicating this sensitively.

- Very important (1)
- Somewhat important (2)
- Neither important nor unimportant (3)
- Somewhat unimportant (4)
- Not at all important (5)

Q7 Any comments or suggested changes to the summary and/or definition? *(for the recommendations we produce from this study)*

________________________________________________________________

________________________________________________________________

________________________________________________________________

________________________________________________________________

________________________________________________________________

| Page Break |  |
| --- | --- |

Q14 **Help individuals prepare for different possible AA outcomes & tolerate uncertainty** - Collaborate with individuals to talk through different possible AA outcomes & preparing for these. May involve: (1) Emotional preparation (e.g. "allow space to tolerate uncertainty"; "how they will cope if [hair loss] returns in the future") (2) Practical preparation (e.g. "having a think about wigs etc")

- Very important (1)
- Somewhat important (2)
- Neither important nor unimportant (3)
- Somewhat unimportant (4)
- Not at all important (5)

Q15 Any comments or suggested changes to the recommendation?

________________________________________________________________

________________________________________________________________

________________________________________________________________

________________________________________________________________

________________________________________________________________

| Page Break |  |
| --- | --- |

Q16 **Help individuals form a clear understanding of AA & make informed decisions** - Help individuals form coherent understanding of AA, via: (1) "possible causes" (e.g. "considering other auto-immune conditions that may need to be addressed") (2) Treatment options (e.g. "various treatments we could try"), including "possible benefits" and side-effects (3) Camouflage options (e.g. "discuss options like scalp micropigmentation, brow microblading, wigs... etc so they feel empowered to make informed decisions" (4) Research opportunities (e.g. "made me aware of ongoing trials etc.").

- Very important (1)
- Somewhat important (2)
- Neither important nor unimportant (3)
- Somewhat unimportant (4)
- Not at all important (5)

Q17 Comments / suggested changes?

________________________________________________________________

________________________________________________________________

________________________________________________________________

________________________________________________________________

________________________________________________________________

| Page Break |  |
| --- | --- |

Q18 **Focus individuals away from self-blaming explanations of AA cause** - Aware of potential for self-blame, regret & frustration in individuals when information & discussion focus on stress-based causes of AA (e.g. "[When] told that stress is the cause of hair loss, [some] try to identify this or start questioning their personality type"). - Note that many people experience high stress but don’t develop AA. - Focus on autoimmune aspects and/or chance causes & support individuals to make sense of their AA.

- Very important (1)
- Somewhat important (2)
- Neither important nor unimportant (3)
- Somewhat unimportant (4)
- Not at all important (5)

Q19 Comments / suggested changes?

________________________________________________________________

________________________________________________________________

________________________________________________________________

________________________________________________________________

________________________________________________________________

| Page Break |  |
| --- | --- |

Q20 **Validate (and explore) the emotional impact of AA** - Taking individuals' emotional difficulties seriously via active listening, "asking open questions about how their alopecia impacts them", empathy & nonjudgement. "Give permission..." that "...all feelings are valid". - Aware of common feelings of grief "of my hair", loss of identity, trauma, isolation, sense of difference, & guilt (e.g. "its not cancer so why am I so upset about hair"). - Acknowledge impact of dismissive responses from others (e.g. "because it's not life threatening, friends can be somewhat dismissive about it"). - Aware that "prior experiences of loss, hopelessness, invalidation can all be reactivated by alopecia".

- Very important (1)
- Somewhat important (2)
- Neither important nor unimportant (3)
- Somewhat unimportant (4)
- Not at all important (5)

Q38 Comments / suggested changes?

________________________________________________________________

________________________________________________________________

________________________________________________________________

________________________________________________________________

________________________________________________________________

| Page Break |  |
| --- | --- |

Q21 Thanks! Now to rank these same recommendations in order of their importance...  (Please drag them into rank order, where 1 = most important)

______ Help individuals form a clear understanding of AA & make informed decisions *(rated as ${Q16/ChoiceGroup/SelectedChoices})* (1)

______ Help individuals prepare for different possible AA outcomes & tolerate uncertainty *(rated as ${Q14/ChoiceGroup/SelectedChoices})* (2)

______ Focus individuals away from self-blaming attributions of AA cause *(rated as ${Q18/ChoiceGroup/SelectedChoices})* (3)

______ Validate (and explore) the emotional impact of AA *(rated as ${Q20/ChoiceGroup/SelectedChoices})* (4)

______ Tactfully manage individuals' expectations of AA outcomes *(rated as ${Q6/ChoiceGroup/SelectedChoices})* (5)

| Page Break |  |
| --- | --- |

Q22 Thanks! Now for the next theme...

End of Block: Theme 1

Start of Block: Theme 2

Q24 **THEME 2** **Supporting psychological and social adjustment to life with AA**

| Page Break |  |
| --- | --- |

Q25 **Nurture individuals' capacity to create positives from life with AA and take helpful perspectives** - Help individuals focus on positives parts of self "aside from just appearance" (e.g. to "realise that my hair didn't define me"). Or help "focus on other things about your appearance - make-up, clothes, fitness so you feel good about the rest of your body". - Help take healthy perspectives, e.g. where appropriate "looking positively at your own good health". - Recognise personal growth, e.g. "many [want to] help others and this also keeps some coming [to peer events]". - Broach gently & at individuals' pace, as otherwise can feel dismissive of current impact.

- Very important (1)
- Somewhat important (2)
- Neither important nor unimportant (3)
- Somewhat unimportant (4)
- Not at all important (5)

Q26 Comments / suggested changes?

________________________________________________________________

________________________________________________________________

________________________________________________________________

________________________________________________________________

________________________________________________________________

| Page Break |  |
| --- | --- |

Q27 **Help individuals prepare for unwanted attention on their appearance** - Acknowledge stigma towards different appearance "in a society that places value on appearance". - Help individuals "manage reactions of others - having prepared responses", "discuss ways to deal with certain situations e.g. questions about alopecia - a brief explanation they can learn, then change the subject, etc.". - "Self-care after [interactions] they know will be challenging."

- Very important (1)
- Somewhat important (2)
- Neither important nor unimportant (3)
- Somewhat unimportant (4)
- Not at all important (5)

Q28 Comments / suggested changes?

________________________________________________________________

________________________________________________________________

________________________________________________________________

________________________________________________________________

________________________________________________________________

| Page Break |  |
| --- | --- |

Q29 **Nurture individuals' acceptance of themselves & the condition** - Help individuals develop - & recognise importance (& challenge) of: (1) Self-acceptance (e.g. "[discussing] how they can fall back in love with themselves whilst adjusting to the new reflection in the mirror”) (2) Acceptance of AA (e.g. "acceptance about the [lack of reliable] cure"). - Help individuals "own the alopecia", by integrating their new appearance into their identity, whether through products/clothing (e.g. headwear) or "embracing bald".

- Very important (1)
- Somewhat important (2)
- Neither important nor unimportant (3)
- Somewhat unimportant (4)
- Not at all important (5)

Q30 Comments / suggested changes?

________________________________________________________________

________________________________________________________________

________________________________________________________________

________________________________________________________________

________________________________________________________________

| Page Break |  |
| --- | --- |

Q31 **Help individuals to adopt authentic strategies to improve quality of life**  - Support individuals to adopt emotional, physical and practical coping strategies that feel authentic: Consider their: (1) Personality & values (e.g. "making light of my alopecia... that suited my personality, it wouldn't suit everyone") (2) Their valued activities (e.g. "as an extremely active individual, the practicalities of wearing a wig were not complimentary to my lifestyle... so I [chose to wear] a headband").

- Very important (1)
- Somewhat important (2)
- Neither important nor unimportant (3)
- Somewhat unimportant (4)
- Not at all important (5)

Q32 Comments / suggested changes?

________________________________________________________________

________________________________________________________________

________________________________________________________________

________________________________________________________________

________________________________________________________________

| Page Break |  |
| --- | --- |

Q33 **Encourage physical self-care**  - Recognise physical self-care as part of healthy adjustment to condition, & its overall health & wellbeing benefits. Could include: (1) "skin and scalp care" (2) Exercise & fitness (3) Diet (4) "adjustments for [physical] comfort… e.g. type of pillow".

- Very important (1)
- Somewhat important (2)
- Neither important nor unimportant (3)
- Somewhat unimportant (4)
- Not at all important (5)

Q37 Comments / suggested changes?

________________________________________________________________

________________________________________________________________

________________________________________________________________

________________________________________________________________

________________________________________________________________

| Page Break |  |
| --- | --- |

Q36 **Support individuals to make decisions about telling/showing others about their AA & how they can do this** - Help individuals decide whether, how & when to share AA diagnosis with others. Enabling them to do so on their terms. Might involve actively: (1) Telling selected people (e.g. "open[ed] up to my family and close friends as well as work, once I did it was no longer a secret that I felt ashamed of..." or (2) 'broadcasting' it (e.g. "when you [feel ready], just tell everyone in one go; social media is a good way - like pulling off a plaster fast." - Recognise challenges like "having to explain it to new people", & anxiety around wearing wigs & accidentally revealing AA.

- Very important (1)
- Somewhat important (2)
- Neither important nor unimportant (3)
- Somewhat unimportant (4)
- Not at all important (5)

Q39 Comments / suggested changes?

________________________________________________________________

________________________________________________________________

________________________________________________________________

________________________________________________________________

________________________________________________________________

| Page Break |  |
| --- | --- |

Q34 Now please rank the recommendations by importance:

______ Help individuals prepare for unwanted attention on their appearance *(${Q27/ChoiceGroup/SelectedChoices})* (1)

______ Help individuals to adopt authentic strategies to improve quality of life *(${Q31/ChoiceGroup/SelectedChoices})* (2)

______ Nurture individuals' acceptance of themselves & the condition *(${Q29/ChoiceGroup/SelectedChoices})* (3)

______ Encourage physical self-care *(${Q33/ChoiceGroup/SelectedChoices})* (4)

______ Nurture individuals' capacity to create positives from life with AA and take helpful perspectives *(${Q25/ChoiceGroup/SelectedChoices})* (5)

______ Support individuals to make decisions about telling/showing others about their AA & how they can do this *(${Q36/ChoiceGroup/SelectedChoices})* (6)

| Page Break |  |
| --- | --- |

Q35 Half way there...

End of Block: Theme 2

Start of Block: Theme 3

Q40 **THEME 3** **Awareness from the supporter**

| Page Break |  |
| --- | --- |

Q41 **Respect and work with individuals' chosen coping strategies (where no clear harm is caused)** - Recognise that "telling someone they don't need to wear a wig or [they] shouldn't hide from people when they are not ready can be very unhelpful and actually very distressing", & "I don’t like being in a forum... where there is pressure to go bald - You need to manage AA in your own way." - Supporting individuals’ chosen practical & emotional coping strategies (e.g. wearing wigs, or focused on hair regrowth). - Focus on whether their strategies aid or hinder quality of life, e.g. if wearing a wig feels necessary to leave house, it may be aiding quality of life.

- Very important (1)
- Somewhat important (2)
- Neither important nor unimportant (3)
- Somewhat unimportant (4)
- Not at all important (5)

Q42 Comments / suggested changes?

________________________________________________________________

________________________________________________________________

________________________________________________________________

________________________________________________________________

________________________________________________________________

| Page Break |  |
| --- | --- |

Q43 **Be aware of any unhelpful coping strategies individuals are using** - Aware of when individuals are adopting understandable yet potentially unhelpful coping strategies long-term; like: (1) Withdrawing from friends & family (2) Denying problem (e.g. "subconsciously hiding it away and 'wish[ing] it away'") (3) Avoiding valued activities or opportunities. - Sensitively helping them become aware of any unhelpful consequences of their strategies

- Very important (1)
- Somewhat important (2)
- Neither important nor unimportant (3)
- Somewhat unimportant (4)
- Not at all important (5)

Q44 Comments / suggested changes?

________________________________________________________________

________________________________________________________________

________________________________________________________________

________________________________________________________________

________________________________________________________________

| Page Break |  |
| --- | --- |

Q45 **Allow for time & fluctuation within individuals while adjusting to life with AA** - Patience towards individuals who may need time to adjust to diagnosis (e.g. "my grief was deep and long before I could begin to "put myself back together""). - Aware of "rollercoaster… [of] cycles of growth, loss, watching, checking, masking etc". - Working at their pace of adjustment & readiness to receive support. - Depending on supporter's role, could involve offering follow-up "review to check up on me" and/or ongoing support to provide "continuity of care". - Keeping support options on agenda throughout timespan of consultations.

- Very important (1)
- Somewhat important (2)
- Neither important nor unimportant (3)
- Somewhat unimportant (4)
- Not at all important (5)

Q46 Comments / suggested changes?

________________________________________________________________

________________________________________________________________

________________________________________________________________

________________________________________________________________

________________________________________________________________

| Page Break |  |
| --- | --- |

Q47 **Hold in mind that individuals' identities may shape their experiences of AA** - Aware that individuals' identities & backgrounds can influence their experience of living with AA, & their support needs, e.g: (1) Ethnicity, race &/or cultural background (e.g. "[In] Asian [culture] a lot of importance is placed on your hair") (2) Gender (e.g. "My hair and my eyelashes, as a woman... when I lost them , I lost my femininity" and "Males, particularly masculine gendered males, cannot wear wigs or eyelashes") (3) Sexuality.

- Very important (1)
- Somewhat important (2)
- Neither important nor unimportant (3)
- Somewhat unimportant (4)
- Not at all important (5)

Q48 Comments / suggested changes?

________________________________________________________________

________________________________________________________________

________________________________________________________________

________________________________________________________________

________________________________________________________________

| Page Break |  |
| --- | --- |

Q51 **Recognise variation between individuals in responding to a diagnosis of AA** - Acknowledge that individuals respond differently to diagnosis. - Aware that extent of hair loss doesn't necessarily predict level of distress ("for some people, a small bald patch is devastating, while others with more severe symptoms adapt quickly"). - Aware that some individuals may mask their suffering ("people with AA are often just putting on a brave face - while feeling really shit!")

- Very important (1)
- Somewhat important (2)
- Neither important nor unimportant (3)
- Somewhat unimportant (4)
- Not at all important (5)

Q52 Comments / suggested changes?

________________________________________________________________

________________________________________________________________

________________________________________________________________

________________________________________________________________

________________________________________________________________

| Page Break |  |
| --- | --- |

Q55 **Cultivate self-awareness of your own assumptions & attitudes about appearance** - "Good psychological support means [you] have worked with your own bias and beauty ideals, [so you] do not unknowingly influence or judge the client or their choices." - Being aware that men with AA can also struggle to adjust (e.g. "[An unhelpful belief is that] men suffer less than women because society got used to male pattern baldness, and they can [all] accept it easily and make jokes about it. False.") - Awareness that individuals don't necessarily want reassurance about their appearance (e.g. "I've been told many times that it's really irritating when people try to reassure").

- Very important (1)
- Somewhat important (2)
- Neither important nor unimportant (3)
- Somewhat unimportant (4)
- Not at all important (5)

Q56 Comments / suggested changes?

________________________________________________________________

________________________________________________________________

________________________________________________________________

________________________________________________________________

________________________________________________________________

| Page Break |  |
| --- | --- |

Q53 Now please rank the recommendations by importance:

______ Recognise variation between individuals in responding to a diagnosis of AA *(${Q51/ChoiceGroup/SelectedChoices})* (1)

______ Allow for time & fluctuation within individuals while adjusting to life with AA *(${Q45/ChoiceGroup/SelectedChoices})* (2)

______ Hold in mind that individuals' identities may shape their experiences *(${Q47/ChoiceGroup/SelectedChoices})* (3)

______ Be aware of any unhelpful coping strategies individuals are using *(${Q43/ChoiceGroup/SelectedChoices})* (4)

______ Respect and work with individuals' chosen coping strategies (where no clear harm is caused) *(${Q41/ChoiceGroup/SelectedChoices})* (6)

______ Cultivate self-awareness of your own assumptions & attitudes about appearance *(${Q55/ChoiceGroup/SelectedChoices})* (7)

| Page Break |  |
| --- | --- |

Q54 Nearly on the home straight now...

End of Block: Theme 3

Start of Block: Theme 4

Q57 **THEME 4** **Delivering support**

| Page Break |  |
| --- | --- |

Q58 **Psychological support should be accessible and flexible to individuals' needs & preferences** - Support from mental health professionals or trained peers should be flexible, to accommodate differences in affected individuals' preferences regarding: (1) Format (i.e. in-person or online; 1-to-1 or group) ,"[support should be] offered in different formats to suit different people" (2) Setting, i.e. in healthcare settings ("[some] prefer to be seen in the hospital where they attend appointments") or community ("medical settings are too cold and daunting.") - "Readily available" support where possible.

- Very important (1)
- Somewhat important (2)
- Neither important nor unimportant (3)
- Somewhat unimportant (4)
- Not at all important (5)

Q59 Comments / suggested changes?

________________________________________________________________

________________________________________________________________

________________________________________________________________

________________________________________________________________

________________________________________________________________

| Page Break |  |
| --- | --- |

Q60 **Psychological support should be offered early on from diagnosis** - Though support should be on agenda throughout individuals' AA journey, it should always be made available from initial consultations. This could help: (1) Speed up adjustment processes (e.g. "I wish I had had psychological and peer support earlier - I think this may have helped me to come to terms with my alopecia at an earlier stage") (2) Provide vital coping strategies (e.g. "I do think some of the negative aspects of the condition wouldn't have had the chance to take hold... healthy coping techniques are essential!")

- Very important (1)
- Somewhat important (2)
- Neither important nor unimportant (3)
- Somewhat unimportant (4)
- Not at all important (5)

Q61 Comments / suggested changes?

________________________________________________________________

________________________________________________________________

________________________________________________________________

________________________________________________________________

________________________________________________________________

| Page Break |  |
| --- | --- |

Q62 **There should be a holistic, multi-support-role & multisector approach to psychological support** - "The best service involves a holistic approach (i.e. addressing all health & wellbeing aspects) with all professionals working together". - Multi-support-role involvement with communication, signposting & shared input & learning, across support roles (e.g. "[As a mental health professional] I find it very helpful to link in with my medical colleagues to help understand the medical context", & "I would like to have been signposted to [charitable] support by a health professional". - Multisector approach, working across medical, charitable & private sectors to provide joined up care.

- Very important (1)
- Somewhat important (2)
- Neither important nor unimportant (3)
- Somewhat unimportant (4)
- Not at all important (5)

Q63 Comments / suggested changes?

________________________________________________________________

________________________________________________________________

________________________________________________________________

________________________________________________________________

________________________________________________________________

| Page Break |  |
| --- | --- |

Q66 **Give time in-session or acknowledge time constraints for individuals to share their experiences** - Wherever possible, give "time [in-session] to express how the alopecia is making them feel e.g. loss of confidence, loss of identity etc". - Given health professionals' time pressures especially in NHS settings, openly naming this challenge, & signposting to support sources (including peer support) where more time can be given.

- Very important (1)
- Somewhat important (2)
- Neither important nor unimportant (3)
- Somewhat unimportant (4)
- Not at all important (5)

Q67 Comments / suggested changes?

________________________________________________________________

________________________________________________________________

________________________________________________________________

________________________________________________________________

________________________________________________________________

| Page Break |  |
| --- | --- |

Q68 **Peer supporters need to skilfully manage the complexities of peer groups**  - Peer facilitators' awareness of potential challenges of peer groups, & ability to manage: (1) Range of members' AA progression and adjustment (e.g. "if someone is really struggling and meeting others who are really positive this can have the opposite effect of being unsupportive and invalidating") (2) Members sharing "horror stories" & high expressed emotion (3) Gender/age/ethnicity imbalances (e.g. "it might not be helpful for a twelve year old to be at a peer support meeting with a group of 50 year old women" (4) Cliques forming. - Need to create safe space by welcoming new members, "giving people plenty of info beforehand so they know what they are coming too, and are prepared".

- Very important (1)
- Somewhat important (2)
- Neither important nor unimportant (3)
- Somewhat unimportant (4)
- Not at all important (5)

Q69 Comments / suggested changes?

________________________________________________________________

________________________________________________________________

________________________________________________________________

________________________________________________________________

________________________________________________________________

| Page Break |  |
| --- | --- |

Q72 Now please rank the recommendations by importance:

______ There should be a holistic, multi-support-role & multisector approach to psychological support *(${Q62/ChoiceGroup/SelectedChoices})* (1)

______ Psychological support should be offered early on from diagnosis *(${Q60/ChoiceGroup/SelectedChoices})* (3)

______ Give time in-session or acknowledge time constraints for individuals to share their experiences *(${Q66/ChoiceGroup/SelectedChoices})* (4)

______ Psychological support should be accessible and flexible to individuals' needs & preferences *(${Q58/ChoiceGroup/SelectedChoices})* (5)

______ Peer supporters need to skilfully manage the complexities of peer groups *(${Q68/ChoiceGroup/SelectedChoices})* (6)

| Page Break |  |
| --- | --- |

Q73 Thank you! Just a very quick one to finish on...

End of Block: Theme 4

Start of Block: Theme 5

Q74 **THEME 4** **Supporting children & young people**

| Page Break |  |
| --- | --- |

Q75 **Consider children & young people’s context**  - Aware of childhood & adolescence context: (1) Still-forming identity (e.g. "interrupt" development of "a strong sense of self") & centrality of feeling accepted by peers (2) Milestones (e.g. "when I left school, started university, travelled etc... there are always aspects of having alopecia that will surprise you") (3) Potential for teasing &/or bullying.

- Very important (1)
- Somewhat important (2)
- Neither important nor unimportant (3)
- Somewhat unimportant (4)
- Not at all important (5)

Q76 Comments / suggested changes?

________________________________________________________________

________________________________________________________________

________________________________________________________________

________________________________________________________________

________________________________________________________________

| Page Break |  |
| --- | --- |

Q77 **Communicate sensitively with children & young people** - Use age-appropriate language (e.g. "keeping literature and the way it is communicated to [children] fun and simple"). - Sensitivity to how overwhelming (or "terrifying”) consultations can be for children or young people.

- Very important (1)
- Somewhat important (2)
- Neither important nor unimportant (3)
- Somewhat unimportant (4)
- Not at all important (5)

Q78 Comments / suggested changes?

________________________________________________________________

________________________________________________________________

________________________________________________________________

________________________________________________________________

________________________________________________________________

| Page Break |  |
| --- | --- |

Q79 **Involve and support the families of children & young people with AA** - Recognise family members’ needs, "involving parents and other family members as appropriate" in supporting the affected child or young person. - Providing "support [for] parents of children suffering with hair loss so they to give them the tools to support their child". - Gently addressing families’ attitudes (explicit or implied) that may impact on the child / young person: "Children [can] suffer from their parent's feelings about their baldness" (e.g. wanting their child to wear a wig and the potential shame this implies).

- Very important (1)
- Somewhat important (2)
- Neither important nor unimportant (3)
- Somewhat unimportant (4)
- Not at all important (5)

Q80 Comments / suggested changes?

________________________________________________________________

________________________________________________________________

________________________________________________________________

________________________________________________________________

________________________________________________________________

| Page Break |  |
| --- | --- |

| Page Break |  |
| --- | --- |

Q87 Now please rank the recommendations by importance:

______ Consider children & young people’s context *(${Q75/ChoiceGroup/SelectedChoices})* (1)

______ Involve and support the families of children & young people with AA *(${Q79/ChoiceGroup/SelectedChoices})* (2)

______ Communicate sensitively with children & young people *(${Q77/ChoiceGroup/SelectedChoices})* (3)

| Page Break |  |
| --- | --- |

Q88 Thank you for taking survey 2. Your involvement is key to shaping the recommendations in a way that reflects the expertise of people who provide and receive support. If you have any queries at all, please don't hesitate to contact me at fabio.zucchelli@uwe.ac.uk Unless you've asked not to receive one, I'll be in touch with the shopping voucher shortly (this may take up to a few days)

End of Block: Theme 5

Start of Block: Block 6

Q74 **THEME 5** **Supporting children & young people**

| Page Break |  |
| --- | --- |

Q75 **Consider children & young people’s context**  - Aware of childhood & adolescence context: (1) Still-forming identity (e.g. "interrupt" development of "a strong sense of self") & centrality of feeling accepted by peers (2) Milestones (e.g. "when I left school, started university, travelled etc... there are always aspects of having alopecia that will surprise you") (3) Potential for teasing &/or bullying.

- Very important (1)
- Somewhat important (2)
- Neither important nor unimportant (3)
- Somewhat unimportant (4)
- Not at all important (5)

Q76 Comments / suggested changes?

________________________________________________________________

________________________________________________________________

________________________________________________________________

________________________________________________________________

________________________________________________________________

| Page Break |  |
| --- | --- |

Q77 **Communicate sensitively with children & young people** - Use age-appropriate language (e.g. "keeping literature and the way it is communicated to [children] fun and simple"). - Sensitivity to how overwhelming (or "terrifying”) consultations can be for children or young people.

- Very important (1)
- Somewhat important (2)
- Neither important nor unimportant (3)
- Somewhat unimportant (4)
- Not at all important (5)

Q78 Comments / suggested changes?

________________________________________________________________

________________________________________________________________

________________________________________________________________

________________________________________________________________

________________________________________________________________

| Page Break |  |
| --- | --- |

Q79 **Involve and support the families of children & young people with AA** - Recognise family members’ needs, "involving parents and other family members as appropriate" in supporting the affected child or young person. - Providing "support [for] parents of children suffering with hair loss so they to give them the tools to support their child". - Gently addressing families’ attitudes (explicit or implied) that may impact on the child / young person: "Children [can] suffer from their parent's feelings about their baldness" (e.g. wanting their child to wear a wig and the potential shame this implies).

- Very important (1)
- Somewhat important (2)
- Neither important nor unimportant (3)
- Somewhat unimportant (4)
- Not at all important (5)

Q80 Comments / suggested changes?

________________________________________________________________

________________________________________________________________

________________________________________________________________

________________________________________________________________

________________________________________________________________

| Page Break |  |
| --- | --- |

Q87 Now please rank the recommendations by importance:

______ Consider children & young people’s context *(${Q75/ChoiceGroup/SelectedChoices})* (1)

______ Involve and support the families of children & young people with AA *(${Q79/ChoiceGroup/SelectedChoices})* (2)

______ Communicate sensitively with children & young people *(${Q77/ChoiceGroup/SelectedChoices})* (3)

| Page Break |  |
| --- | --- |

Q88 Thank you for taking survey 2. Your involvement is key to shaping the recommendations in a way that reflects the expertise of people who provide and receive support. If you have any queries at all, please don't hesitate to contact me at fabio.zucchelli@uwe.ac.uk Unless you've asked not to receive one, I'll be in touch with the shopping voucher shortly (this may take up to a few days)

End of Block: Theme 5

AA Delphi Survey 3

Start of Block: Sorting

Q2 Please drag each recommendation into one of the 3 boxes that you think best describes its relevance and/or suitability to your role. If you would like a reminder of exactly what each recommendation means, please click on the hyperlinked text at the bottom of the screen.  *Category*: **Affected individuals' perception of AA: It's causes, outcomes and impact**

| Directly relevant to/suitable for my role | Partly relevant to/suitable for my role | Not relevant to/suitable for my role |
| --- | --- | --- |
| ______ Be sensitive to how individuals may perceive suggestions of stress-based causes of AA <em>(changed from "Focus individuals away from self-blaming attributions of AA")</em> (1) | ______ Be sensitive to how individuals may perceive suggestions of stress-based causes of AA <em>(changed from "Focus individuals away from self-blaming attributions of AA")</em> (1) | ______ Be sensitive to how individuals may perceive suggestions of stress-based causes of AA <em>(changed from "Focus individuals away from self-blaming attributions of AA")</em> (1) |
| ______ Help individuals prepare for different possible AA outcomes & tolerate uncertainty (2) | ______ Help individuals prepare for different possible AA outcomes & tolerate uncertainty (2) | ______ Help individuals prepare for different possible AA outcomes & tolerate uncertainty (2) |
| ______ Help individuals form a clear understanding of AA & make informed decisions (3) | ______ Help individuals form a clear understanding of AA & make informed decisions (3) | ______ Help individuals form a clear understanding of AA & make informed decisions (3) |
| ______ Validate (and explore) the emotional impact of AA (4) | ______ Validate (and explore) the emotional impact of AA (4) | ______ Validate (and explore) the emotional impact of AA (4) |
| ______ Tactfully manage individuals' expectations of AA outcomes (5) | ______ Tactfully manage individuals' expectations of AA outcomes (5) | ______ Tactfully manage individuals' expectations of AA outcomes (5) |

Q5 *Optional:*  Any comments on items you selected as "Partly" or "Not relevant / suitable"? (e.g. whether there are any modified versions of the item that might be more relevant / suitable?)

________________________________________________________________

________________________________________________________________

________________________________________________________________

________________________________________________________________

________________________________________________________________

| Page Break |  |
| --- | --- |

Q3 **Supporting psychological and social adjustment to life with AA**

| Directly relevant to/suitable for my role | Partly relevant to/suitable for my role | Not relevant to/suitable for my role |
| --- | --- | --- |
| ______ Nurture individuals' capacity to create positives from life with AA and take helpful perspectives (1) | ______ Nurture individuals' capacity to create positives from life with AA and take helpful perspectives (1) | ______ Nurture individuals' capacity to create positives from life with AA and take helpful perspectives (1) |
| ______ Nurture individuals' acceptance of themselves & the condition (2) | ______ Nurture individuals' acceptance of themselves & the condition (2) | ______ Nurture individuals' acceptance of themselves & the condition (2) |
| ______ Help individuals prepare for unwanted attention on their appearance (3) | ______ Help individuals prepare for unwanted attention on their appearance (3) | ______ Help individuals prepare for unwanted attention on their appearance (3) |
| ______ Help individuals to adopt authentic strategies to improve quality of life (4) | ______ Help individuals to adopt authentic strategies to improve quality of life (4) | ______ Help individuals to adopt authentic strategies to improve quality of life (4) |
| ______ Support individuals to make decisions about telling/showing others about their AA & how they can do this (5) | ______ Support individuals to make decisions about telling/showing others about their AA & how they can do this (5) | ______ Support individuals to make decisions about telling/showing others about their AA & how they can do this (5) |

Q6 *Optional:*  Any comments on items you selected as "Partly" or "Not relevant / suitable"? (e.g. whether there are any modified versions of the item that might be more relevant / suitable?)

________________________________________________________________

________________________________________________________________

________________________________________________________________

________________________________________________________________

________________________________________________________________

| Page Break |  |
| --- | --- |

Q4 **Awareness from the supporter**

| Directly relevant to/suitable for my role | Partly relevant to/suitable for my role | Not relevant to/suitable for my role |
| --- | --- | --- |
| ______ Respect and work with individuals' chosen coping strategies (where no clear harm is caused) (1) | ______ Respect and work with individuals' chosen coping strategies (where no clear harm is caused) (1) | ______ Respect and work with individuals' chosen coping strategies (where no clear harm is caused) (1) |
| ______ Be aware of any unhelpful coping strategies individuals are using (2) | ______ Be aware of any unhelpful coping strategies individuals are using (2) | ______ Be aware of any unhelpful coping strategies individuals are using (2) |
| ______ Allow for time & fluctuation within individuals while adjusting to life with AA (3) | ______ Allow for time & fluctuation within individuals while adjusting to life with AA (3) | ______ Allow for time & fluctuation within individuals while adjusting to life with AA (3) |
| ______ Recognise variation between individuals in responding to a diagnosis of AA (4) | ______ Recognise variation between individuals in responding to a diagnosis of AA (4) | ______ Recognise variation between individuals in responding to a diagnosis of AA (4) |
| ______ Hold in mind that individuals' identities may shape their experiences (5) | ______ Hold in mind that individuals' identities may shape their experiences (5) | ______ Hold in mind that individuals' identities may shape their experiences (5) |
| ______ Cultivate self-awareness of your own assumptions & attitudes about appearance (6) | ______ Cultivate self-awareness of your own assumptions & attitudes about appearance (6) | ______ Cultivate self-awareness of your own assumptions & attitudes about appearance (6) |

Q7 *Optional:*  Any comments on items you selected as "not relevant / suitable"? (e.g. whether there are any modified versions of the item that might be more relevant / suitable?)

________________________________________________________________

________________________________________________________________

________________________________________________________________

________________________________________________________________

________________________________________________________________

| Page Break |  |
| --- | --- |

Q8 **Supporting children & young people**

| Directly relevant to/suitable for my role | Partly relevant to/suitable for my role | Not relevant to/suitable for my role |
| --- | --- | --- |
| ______ Consider children & young people’s context (1) | ______ Consider children & young people’s context (1) | ______ Consider children & young people’s context (1) |
| ______ Communicate sensitively with children & young people (2) | ______ Communicate sensitively with children & young people (2) | ______ Communicate sensitively with children & young people (2) |
| ______ Involve and support the families of children & young people with AA (3) | ______ Involve and support the families of children & young people with AA (3) | ______ Involve and support the families of children & young people with AA (3) |

Q9 *Optional:*  Any comments on items you selected as "not relevant / suitable"? (e.g. whether there are any modified versions of the item that might be more relevant / suitable?)

________________________________________________________________

________________________________________________________________

________________________________________________________________

________________________________________________________________

________________________________________________________________

End of Block: Sorting

Start of Block: Preferences

Q10 Do you have any advice on how best to present the recommendations to those in your role? (i.e. medics, mental health professionals, peer supporters or trichologists). This might be in terms of: Key events / platforms in which to share the recommendations Level of detail given for each item Items presented in priority order? Anonymised quotes from experts used to help explain items?

Any other advice is also very welcome!

________________________________________________________________

________________________________________________________________

________________________________________________________________

________________________________________________________________

________________________________________________________________

End of Block: Preferences

Start of Block: Thank you
