# Supplementary material for: Developing recommendations for promoting wellbeing in individuals with alopecia areata: a modified Delphi study
Source: BMJ Open. 2026 Feb 10;16(2):e094491. doi: 10.1136/bmjopen-2024-094491 (PMC12911720; doi:10.1136/bmjopen-2024-094491)
Supplement: Supplementary file 3 [file bmjopen-2024-094491.reviewer_comments.pdf]

PEER REVIEW HISTORY

BMJ Open publishes all reviews undertaken for accepted manuscripts. Reviewers are asked to complete a checklist review form and are provided with free text boxes to elaborate on their assessment. These free text comments are reproduced below.

ARTICLE DETAILS

Title (Provisional)

Developing recommendations for promoting wellbeing in individuals with Alopecia Areata: A modified Delphi study

Authors

Zucchelli, Fabio; Montgomery, Kerry

VERSION 1 - REVIEW

Reviewer 1

Name Huck, Garrett

Affiliation University of Wisconsin-Madison, Department of Rehabilitation Psychology and Special Education

Date 06-Mar-2025

COI None

Thank you for the opportunity to review this interesting and valuable study. I think it answers valid questions, as it is suggested that providers should be more attuned to, and address, the psychosocial needs of people living with AA. However, it is not always clear how this can or should be done. The suggestions in this article make sense to me.

While I believe the proper information is there, the methodology section felt somewhat long, dense, and tangential to me. It may be beneficial to make this section more concise and to the point for each step of the modified Delphi study. the tables being mislabeled was very confusing as well. Be sure to double check that and fix the problem.

In the intro, 2 studies on meditation and one on hypnosis were mentioned as psychosocial intervention studies. I believe there are more. I am not sure if there was a reason that only three are mentioned. Perhaps spend some time double-checking into this.

Perhaps in the discussion section, the authors could consider how we can actually go about establishing standards of practice amongst multidisciplinary providers. In other words, how can the word get out about the roles of different providers?

****** The reviewer provided a marked copy with additional comments. Please contact the publisher for full details. ******

**Reviewer**	**2**
**Name**	**Reich , Adam**
**Affiliation**	**University of Rzeszów College of Medical Sciences**
**Date**	**02-Aug-2025**
**COI**	**None**

The authors aimed to develop recommendations for improving the well-being of patients with alopecia areata. Potentially, the idea is worth considering; however, it cannot be implemented without taking into account the available treatment options. The entire manuscript is also lengthy and difficult to follow. The elaborated recommendations are not wrong, but are not specific for alopecia areata and are true for any chronic (skin) condition. The topic is also of interest for a rather small group of specialists, and as such, the manuscript is better suited for a more specialized journal.

VERSION 1 - AUTHOR RESPONSE

Reviewer: 1
Dr. Garrett Huck, University of Wisconsin-Madison

Comments to the Author:
Thank you for the opportunity to review this interesting and valuable study. I think it answers valid questions, as it is suggested that providers should be more attuned to, and address, the psychosocial needs of people living with AA. However, it is not always clear how this can or should be done. The suggestions in this article make sense to me.

While I believe the proper information is there,
10. The methodology section felt somewhat long, dense, and tangential to me. It may be beneficial to make this section more concise and to the point for each step of the modified Delphi study.

We certainly take this point. Much of the length and density can be explained by BMJ Open's requirement that authors of Delphi studies adhere to the ACCORD Accurate Consensus Reporting Document. This document places particular emphasis on providing comprehensive detail of the methods for transparency and to aid replicability. Examples of points to be covered that add a lot of detail are "If modifications were made to the method in its original form, provide a detailed explanation of how the method was adjusted and why this was necessary for the purpose of your consensus-based study", and "Describe the recruitment process (how panellists were invited to participate). Include communication/advertisement method(s) and locations, numbers of invitations sent, and whether there was centralised oversight of invitations or if panellists were asked/allowed to suggest other members of the panel."

However, we acknowledge that in an effort to ensure we have covered all of these criteria, we may have gone overboard and created an unwieldy methods section. So we have reviewed and edited this section to hopefully remove any information that isn't critical to the ACCORD document. After these edits, we have reduced the Methods section by 13%.

11. The tables being mislabeled was very confusing as well. Be sure to double check that and fix the problem.

Apologies for this error and the frustration it must have caused. We have corrected the label for Table 4.

12. In the intro, 2 studies on meditation and one on hypnosis were mentioned as psychosocial intervention studies. I believe there are more. I am not sure if there was a reason that only three are mentioned. Perhaps spend some time double-checking into this.

We acknowledge that there were other published papers on the topic. We chose the three cited articles as the highest quality studies that can be said to offer positive empirical evidence. For example, other unmentioned studies include:
(1) An uncontrolled prospective study published in a letter to the editor: Harrison, P. V., & Stepanek, P. (1991). Hypnotherapy for alopecia areata. British Journal of Dermatology, 124(5), 509-510; and
(2) one study that applied brief 'behavioural health' intervention and found non-significant effects compared to the control group: Gorbatenko-Roth, K., Hodges, J. S., Lifson, D., Golm, M., Kranz, D., Windenburg, D., & Hordinsky, M. (2021). Integrating colocated behavioral health care into a dermatology clinic: a prospective randomized controlled treatment pilot study in patients with alopecia areata. Journal of the American Academy of Dermatology, 84(5), 1487-1489.

In writing the report, we used the following systematic reviews to source these papers:
(a) Tkachenko, E., Okhovat, J. P., Manjaly, P., Huang, K. P., Senna, M. M., & Mostaghimi, A. (2023). Complementary and alternative medicine for alopecia areata: A systematic review. Journal of the American Academy of Dermatology, 88(1), 131-143. And
(b) Maloh, J., Engel, T., Natarelli, N., Nong, Y., Zufall, A., & Sivamani, R. K. (2023). Systematic review of psychological interventions for quality of life, mental health, and hair growth in alopecia areata and scarring alopecia. Journal of clinical medicine, 12(3), 964.
That being said Willemsen and colleagues did conduct more studies on hypnotherapy. But for parsimony we just cited the most up-to-date study (2011). We have therefore added the 2006 paper for completeness.
https://www.sciencedirect.com/science/article/pii/S0190962205030215?casa_token=nY7cZEdXgAUAAAAA:YaoKX-_bzTirdGYCNEos11f7YfZpw11V-2OxorhbA4mwrEoW3cXFBvgIh1CASJPw2wXxJL9CPww

13. Perhaps in the discussion section, the authors could consider how we can actually go about establishing standards of practice amongst multidisciplinary providers. In other words, how can the word get out about the roles of different providers?

Thank you for this suggestion. We have added the following text: "This can be achieved by using the recommendations as a basis for educational resources across the different professional organisations (e.g. British Association for Counselling and Psychotherapy; The

Primary Care Dermatology Society), and incorporating the recommendations into existing professional guidelines (e.g. British Association of Dermatologists' living guidelines for managing AA)."

14. Remove inappropriate capitalisations.

We have removed the capitalisations for Alopecia Areata, Dermatologist, General Practitioner and Trichologist.

15. Going back to my prior comment on there being more interventions, this [peer support] is an example for which I believe there is existing research.

Thank you for raising this. We have added the only paper that came up in our literature search to the main text: "Qualitative research suggests that women who used an online peer support forum found the process validating and normalising, though they also reported unhelpful sharing of inaccurate health information (Iliffe & Thompson, 2019). To our knowledge, however, there is no published research on in-person peer support for AA."

16. I believe that individual differences such as time since onset, age, etc may influence perceptions of experience and intervention. I believe it is important to note that an inability to account for such factors is a limitation.

Thank you for this helpful feedback. We have added the following: "It is also important to recognise the potential influence in the expert by experience group of factors like age, time since AA onset, and type of wellbeing support received on their perspectives on optimal wellbeing promotion. The study design precluded closer examination of these factors."

Please see attached additional comments.

Reviewer: 2
Dr. Adam Reich , University of Rzeszów College of Medical Sciences

Comments to the Author:
The authors aimed to develop recommendations for improving the well-being of patients with alopecia areata. Potentially, the idea is worth considering; however,
17. It cannot be implemented without taking into account the available treatment options.

We're unsure whether 'treatment options' refers to medical and/or psychological treatment options. To provide more practically oriented direction in the context of treatment pathways, to implement the recommendations, we have added the following: "This can be achieved by using the recommendations as a basis for educational resources across the different professional organisations (e.g. British Association for Counselling and Psychotherapy; The Primary Care Dermatology Society), and incorporating the recommendations into existing professional guidelines (e.g. British Association of Dermatologists' living guidelines for managing AA)."

18. The entire manuscript is also lengthy and difficult to follow.

We certainly take this point. Much of the length and density can be explained by BMJ Open's requirement that authors of Delphi studies adhere to the ACCORD Accurate Consensus

Reporting Document. This document places particular emphasis on providing comprehensive detail of the methods for transparency and to aid replicability. Examples of points to be covered that add a lot of detail are “If modifications were made to the method in its original form, provide a detailed explanation of how the method was adjusted and why this was necessary for the purpose of your consensus-based study”, and “Describe the recruitment process (how panellists were invited to participate). Include communication/advertisement method(s) and locations, numbers of invitations sent, and whether there was centralised oversight of invitations or if panellists were asked/allowed to suggest other members of the panel.”

However, we acknowledge that in an effort to ensure we have covered all of these criteria, we may have gone overboard and created an unwieldy methods section. So we have reviewed and edited this section to hopefully remove any information that isn’t critical to the ACCORD document. After these edits, we have reduced the Methods section by 13%.
